# (*E*)-1-(2,4-Dinitro­phen­yl)-2-(2-fluoro­benzyl­idene)hydrazine

**DOI:** 10.1107/S1600536811014383

**Published:** 2011-04-22

**Authors:** Jerry P. Jasinski, Adam N. Braley, C. S. Chidan Kumar, H. S. Yathirajan, A. N. Mayekar

**Affiliations:** a Department of Chemistry, Keene State College, 229 Main Street, Keene, NH 03435-2001, USA; b Department of Studies in Chemistry, University of Mysore, Manasagangotri, Mysore 570 006, India; c SeQuent Scientific Ltd, Baikampady, New Mangalore 575 011, India

## Abstract

In the title compound, C_13_H_9_FN_4_O_4_, the dihedral angle between the mean planes of the two benzene rings of the nearly planar mol­ecule is 6.6 (9)°. The dihedral angles between the mean planes of the benzene ring and its two attached nitro groups are 6.7 (7) and 7.2 (9)°. Crystal packing is stabilized by N—H⋯O hydrogen bonds, weak C—H⋯O and C—H⋯F inter­molecular inter­actions and centroid–centroid π-ring stacking inter­actions.

## Related literature

For Schiff base propeties, see: Liang (2007 ▶). For nonlinear optical and crystalline properties, see: Baughman *et al.* (2004 ▶). For DNA-damaging and mutagenic agents, see: Okabe *et al.* (1993 ▶). For related structures, see: Bolte & Dill (1998 ▶); Shan *et al.* (2002 ▶); Fan *et al.* (2004 ▶); Motherwell & Ramsay, (2007 ▶); Shi *et al.* (2008 ▶); Ji *et al.* (2010 ▶); Kia *et al.* (2009 ▶); Jasinski *et al.* (2010 ▶).
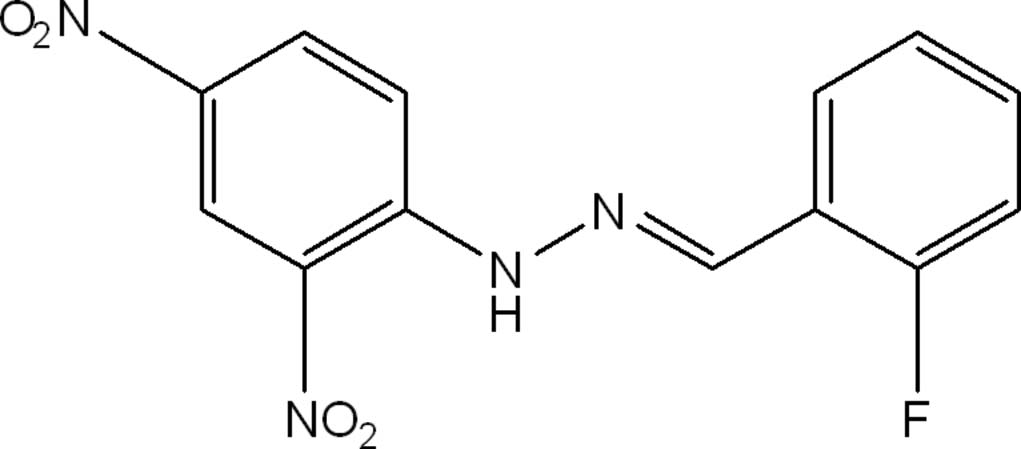


## Experimental

### 

#### Crystal data

C_13_H_9_FN_4_O_4_
*M* *_r_* = 304.24
Triclinic, 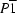

*a* = 7.0961 (8) Å
*b* = 8.2714 (9) Å
*c* = 11.7230 (8) Å
α = 88.614 (7)°
β = 80.544 (8)°
γ = 71.368 (10)°
*V* = 642.86 (11) Å^3^
*Z* = 2
Mo *K*α radiation
μ = 0.13 mm^−1^
*T* = 173 K
0.20 × 0.18 × 0.15 mm

#### Data collection

Oxford Diffraction Xcalibur Eos Gemini diffractometer
Absorption correction: multi-scan (*CrysAlis RED*; Oxford Diffraction, 2007 ▶) *T*_min_ = 0.967, *T*_max_ = 1.000
6346 measured reflections
3466 independent reflections
2802 reflections with *I* > 2σ(*I*)
*R*_int_ = 0.016

#### Refinement

*R*[*F* ^2^ > 2σ(*F* ^2^)] = 0.045
*wR*(*F* ^2^) = 0.143
*S* = 1.09
3466 reflections
199 parameters
H-atom parameters constrained
Δρ_max_ = 0.31 e Å^−3^
Δρ_min_ = −0.17 e Å^−3^

### 

Data collection: *CrysAlis PRO* (Oxford Diffraction, 2007 ▶); cell refinement: *CrysAlis PRO*; data reduction: *CrysAlis RED* (Oxford Diffraction, 2007 ▶); program(s) used to solve structure: *SHELXS97* (Sheldrick, 2008 ▶); program(s) used to refine structure: *SHELXL97* (Sheldrick, 2008 ▶); molecular graphics: *SHELXTL* (Sheldrick, 2008 ▶); software used to prepare material for publication: *SHELXTL*.

## Supplementary Material

Crystal structure: contains datablocks global, I. DOI: 10.1107/S1600536811014383/ng5151sup1.cif

Structure factors: contains datablocks I. DOI: 10.1107/S1600536811014383/ng5151Isup2.hkl

Additional supplementary materials:  crystallographic information; 3D view; checkCIF report

## Figures and Tables

**Table 1:** Hydrogen-bond geometry (Å, °)

*D*—H⋯*A*	*D*—H	H⋯*A*	*D*⋯*A*	*D*—H⋯*A*
N2—H2*A*⋯O1	0.88	2.02	2.6317 (15)	126
N2—H2*A*⋯O1^i^	0.88	2.51	3.3424 (15)	158
C2—H2*B*⋯F1^ii^	0.95	2.45	3.3386 (17)	156
C3—H3*A*⋯O4^iii^	0.95	2.48	3.3177 (19)	148
C5—H5*A*⋯O3^iv^	0.95	2.43	3.2694 (17)	148

Symmetry codes: (i) 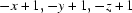; (ii) 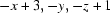; (iii) 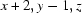; (iv) 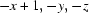.

**Table 2:** *Cg*⋯*Cg* π-ring stacking inter­actions *Cg*1 and *Cg*2 are the centroids of rings C1–C6 and C8–C13, respectively.

*CgI*⋯*CgJ*	*Cg*⋯*Cg* (Å)	*CgI*_Perp (Å)	*CgJ*_Perp (Å)
*Cg*1⋯*Cg*2^i^	3.6916 (10)	−3.4632 (6)	3.3267 (5)
*Cg*2⋯*Cg*1^ii^	3.6916 (10)	3.3267 (5)	−3.4632 (6)

Symmetry codes: (i) 1 + *x*, *y*, *z*; (ii) −1 + *x*, *y*, *z*.

**Fig. 1.**
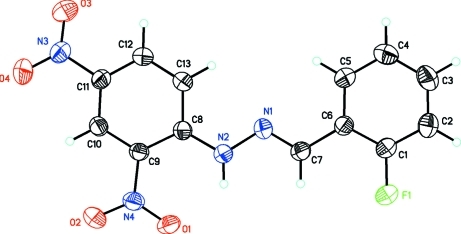
Molecular structure of the title compound showing the atom labeling scheme and 50% probability displacement ellipsoids.

**Fig. 2.**
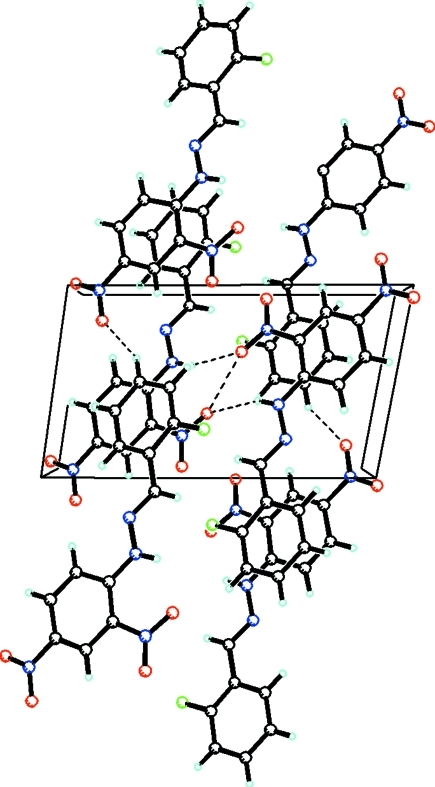
Packing diagram of the title compound viewed down the b axis. Dashed lines indicate N—H···O hydrogen bonds and weak N—H···O intermolecular interactions.

## supplementary crystallographic information

### Comment

Schiff bases and their complexes are widely used in the fields of biology, catalysis etc. (Liang, 2007). Especially, the dinitrophenyl hydrazones exhibit good nonlinear optical (NLO) and crystalline properties (Baughman *et al.*, 2004) and are found to have versatile coordinating abilities towards different metal ions. In addition, some 2,4-dinitrophenyl hydrazone derivatives have been shown to be potentially DNA-damaging and mutagenic agents (Okabe *et al.*, 1993).  As a result of their significant molecular nonlinearities many x-ray structural studies of 2,4-dinitrophenylhydrazones have been reported. Among them, the most closely related structures are (E)-p-methoxy-acetophenone 2,4-dinitrophenylhydrazone (Bolte & Dill, 1998), acetophenone (2,4-dinitrophenyl)hydrazone (Shan *et al.*, 2002), 3-chloroacetophenone 2,4-dintrophenyl- hydrazone (Fan *et al.*, 2004), 2,4-dihydroxyacetophenone 2,4-dinitrophenylhydrazone (Baughman *et al.*, 2004), syn-acetophenone (2,4-dinitrophenyl) hydrazone (Motherwell & Ramsay, 2007), 1-(2-chlorobenzylidene)-2-(2,4-dinitrophenyl)hydrazine (Shi *et al.*, 2008), N-(2,4-dinitrophenyl)-N'-(1-p-tolylethylidene) hydrazine (Kia *et al.*, 2009), N-(2,4-dinitrophenyl)-N'-(1-phenylethylidene)hydrazine (Ji *et al.*, 2010) and (1E)-1-(3-bromophenyl)ethanone 2,4-dinitrophenylhydrazone (Jasinski *et al.*, 2010).  In view of the importance of 2,4-dinitrophenylhydrazones, this paper reports the crystal structure of the title compound, C_13_H_9_FN_4_O_4_, (I).

In the title compound the dihedral angle between the mean planes of the two benzene rings of a nearly planar molecule is 6.69°, (Fig. 2).  The dihedral angle between the mean planes of the benzene ring and its two bonded nitro groups are 6.7 (7)° and 7.2 (9)°, respectively. Crystal packing is stabilized by N—H···O hydrogen bonds (Fig. 3), weak C—H···O intermolecular interactions and Cg—Cg π-ring stacking interactions (Table 2).

### Experimental

A mixture of 2,4-dinitrophenylhydrazine (1.98 g) and 2-fluorobenzaldehyde (1.24 g) was dissolved in methanol and refluxed for about 6h. The precipitate formed was filtered, dried and recrystallized in ethlyacetate. X-ray quality crystals of the title compound (I), were obtained after three days by the slow evaporation of a 1:1 mixture of dimethylformamide and pyridine at room temperature. (mp: 502 - 505 K).

### Refinement

The parameters of all the H atoms have been constrained within the riding atom approximation. C—H bond lengths were constrained to 0.95 Å for aryl atoms, *U*_iso_(H) = 1.18–1.20*U*_eq_(C_aryl_). N—H bond lengths were constrained to 0.88 Å, *U*_iso_(H) = 1.20*U*_eq_(N).

### Crystal data

**Table:**

C_13_H_9_FN_4_O_4_	*Z* = 2
*M**_r_* = 304.24	*F*(000) = 312
Triclinic, *P*1	*D*_x_ = 1.572 Mg m^−^^3^
Hall symbol: -P 1	Mo *K*α radiation, λ = 0.71073 Å
*a* = 7.0961 (8) Å	Cell parameters from 3528 reflections
*b* = 8.2714 (9) Å	θ = 3.1–32.2°
*c* = 11.7230 (8) Å	µ = 0.13 mm^−^^1^
α = 88.614 (7)°	*T* = 173 K
β = 80.544 (8)°	Block, orange-red
γ = 71.368 (10)°	0.20 × 0.18 × 0.15 mm
*V* = 642.86 (11) Å^3^	

### Data collection

**Table:**

Oxford Diffraction Xcalibur Eos Gemini diffractometer	3466 independent reflections
Radiation source: Enhance (Mo) X-ray Source	2802 reflections with *I* > 2σ(*I*)
graphite	*R*_int_ = 0.016
Detector resolution: 16.1500 pixels mm^-1^	θ_max_ = 29.1°, θ_min_ = 3.1°
φ and ω scans	*h* = −9→8
Absorption correction: multi-scan (*CrysAlis RED*; Oxford Diffraction, 2007)	*k* = −11→11
*T*_min_ = 0.967, *T*_max_ = 1.000	*l* = −16→15
6346 measured reflections	

### Refinement

**Table:**

Refinement on *F*^2^	Primary atom site location: structure-invariant direct methods
Least-squares matrix: full	Secondary atom site location: difference Fourier map
*R*[*F*^2^ > 2σ(*F*^2^)] = 0.045	Hydrogen site location: inferred from neighbouring sites
*wR*(*F*^2^) = 0.143	H-atom parameters constrained
*S* = 1.09	*w* = 1/[σ^2^(*F*_o_^2^) + (0.0733*P*)^2^ + 0.0845*P*] where *P* = (*F*_o_^2^ + 2*F*_c_^2^)/3
3466 reflections	(Δ/σ)_max_ < 0.001
199 parameters	Δρ_max_ = 0.31 e Å^−^^3^
0 restraints	Δρ_min_ = −0.17 e Å^−^^3^

### Special details

**Table:**

Geometry. All esds (except the esd in the dihedral angle between two l.s. planes) are estimated using the full covariance matrix. The cell esds are taken into account individually in the estimation of esds in distances, angles and torsion angles; correlations between esds in cell parameters are only used when they are defined by crystal symmetry. An approximate (isotropic) treatment of cell esds is used for estimating esds involving l.s. planes.
Refinement. Refinement of *F*^2^ against ALL reflections. The weighted *R*-factor *wR* and goodness of fit *S* are based on *F*^2^, conventional *R*-factors *R* are based on *F*, with *F* set to zero for negative *F*^2^. The threshold expression of *F*^2^ > σ(*F*^2^) is used only for calculating *R*-factors(gt) *etc*. and is not relevant to the choice of reflections for refinement. *R*-factors based on *F*^2^ are statistically about twice as large as those based on *F*, and *R*- factors based on ALL data will be even larger.

### Fractional atomic coordinates and isotropic or equivalent isotropic displacement parameters (Å^2^)

**Table:**

	*x*	*y*	*z*	*U*_iso_*/*U*_eq_	
F1	1.25967 (13)	0.08268 (12)	0.46347 (7)	0.0542 (3)	
O1	0.33061 (16)	0.49706 (16)	0.46244 (9)	0.0576 (3)	
O2	0.04251 (15)	0.62057 (14)	0.41195 (9)	0.0539 (3)	
O3	0.0526 (2)	0.21724 (19)	−0.01824 (12)	0.0791 (4)	
O4	−0.15820 (15)	0.42413 (15)	0.08977 (10)	0.0561 (3)	
N1	0.79790 (15)	0.14775 (14)	0.28682 (9)	0.0377 (2)	
N2	0.61603 (16)	0.26801 (15)	0.32453 (9)	0.0391 (3)	
H2A	0.5959	0.3291	0.3884	0.047*	
N3	0.00969 (19)	0.32023 (16)	0.06233 (11)	0.0443 (3)	
N4	0.20937 (16)	0.51447 (14)	0.39505 (10)	0.0393 (3)	
C1	1.2885 (2)	−0.02327 (17)	0.37123 (11)	0.0381 (3)	
C2	1.4763 (2)	−0.1424 (2)	0.33969 (14)	0.0499 (3)	
H2B	1.5823	−0.1511	0.3819	0.060*	
C3	1.5057 (2)	−0.2487 (2)	0.24485 (15)	0.0534 (4)	
H3A	1.6336	−0.3321	0.2214	0.064*	
C4	1.3510 (2)	−0.23481 (18)	0.18385 (13)	0.0482 (3)	
H4A	1.3727	−0.3086	0.1187	0.058*	
C5	1.1651 (2)	−0.11396 (17)	0.21735 (11)	0.0404 (3)	
H5A	1.0595	−0.1052	0.1747	0.049*	
C6	1.12930 (18)	−0.00419 (15)	0.31287 (10)	0.0332 (3)	
C7	0.93392 (18)	0.12582 (16)	0.34949 (11)	0.0358 (3)	
H7A	0.9091	0.1924	0.4185	0.043*	
C8	0.46749 (17)	0.29040 (15)	0.26058 (10)	0.0329 (3)	
C9	0.26980 (18)	0.40469 (15)	0.29198 (10)	0.0324 (3)	
C10	0.12047 (18)	0.41764 (14)	0.22616 (10)	0.0336 (3)	
H10A	−0.0115	0.4954	0.2487	0.040*	
C11	0.16630 (19)	0.31661 (15)	0.12820 (11)	0.0345 (3)	
C12	0.3609 (2)	0.20726 (17)	0.09136 (11)	0.0396 (3)	
H12A	0.3909	0.1412	0.0216	0.048*	
C13	0.50799 (19)	0.19515 (17)	0.15534 (11)	0.0390 (3)	
H13A	0.6408	0.1213	0.1290	0.047*	

### Atomic displacement parameters (Å^2^)

**Table:**

	*U*^11^	*U*^22^	*U*^33^	*U*^12^	*U*^13^	*U*^23^
F1	0.0485 (5)	0.0679 (6)	0.0446 (5)	−0.0099 (4)	−0.0184 (4)	−0.0156 (4)
O1	0.0428 (6)	0.0767 (7)	0.0512 (6)	−0.0123 (5)	−0.0111 (5)	−0.0292 (5)
O2	0.0424 (6)	0.0553 (6)	0.0517 (6)	0.0007 (5)	−0.0030 (4)	−0.0189 (5)
O3	0.0686 (8)	0.0871 (9)	0.0790 (9)	−0.0060 (7)	−0.0370 (7)	−0.0388 (7)
O4	0.0380 (5)	0.0613 (7)	0.0664 (7)	−0.0057 (5)	−0.0209 (5)	−0.0032 (5)
N1	0.0290 (5)	0.0430 (6)	0.0384 (6)	−0.0080 (4)	−0.0046 (4)	−0.0036 (4)
N2	0.0292 (5)	0.0480 (6)	0.0373 (5)	−0.0077 (4)	−0.0053 (4)	−0.0108 (4)
N3	0.0440 (6)	0.0463 (6)	0.0455 (6)	−0.0131 (5)	−0.0174 (5)	−0.0022 (5)
N4	0.0348 (5)	0.0427 (6)	0.0392 (6)	−0.0121 (4)	−0.0013 (4)	−0.0110 (4)
C1	0.0385 (6)	0.0426 (6)	0.0338 (6)	−0.0116 (5)	−0.0102 (5)	−0.0005 (5)
C2	0.0391 (7)	0.0540 (8)	0.0529 (8)	−0.0048 (6)	−0.0174 (6)	0.0011 (6)
C3	0.0407 (7)	0.0461 (7)	0.0613 (9)	0.0020 (6)	−0.0060 (6)	−0.0041 (7)
C4	0.0529 (8)	0.0415 (7)	0.0455 (8)	−0.0103 (6)	−0.0031 (6)	−0.0091 (6)
C5	0.0408 (7)	0.0430 (7)	0.0393 (7)	−0.0136 (5)	−0.0107 (5)	−0.0040 (5)
C6	0.0317 (6)	0.0357 (6)	0.0329 (6)	−0.0113 (5)	−0.0061 (4)	0.0007 (4)
C7	0.0333 (6)	0.0418 (6)	0.0331 (6)	−0.0129 (5)	−0.0054 (5)	−0.0042 (5)
C8	0.0286 (5)	0.0363 (6)	0.0339 (6)	−0.0108 (5)	−0.0044 (4)	−0.0034 (4)
C9	0.0313 (6)	0.0335 (5)	0.0318 (6)	−0.0103 (4)	−0.0027 (4)	−0.0058 (4)
C10	0.0298 (6)	0.0313 (5)	0.0382 (6)	−0.0076 (4)	−0.0056 (4)	−0.0021 (4)
C11	0.0354 (6)	0.0335 (6)	0.0363 (6)	−0.0104 (5)	−0.0115 (5)	−0.0006 (5)
C12	0.0394 (7)	0.0403 (6)	0.0361 (6)	−0.0071 (5)	−0.0077 (5)	−0.0092 (5)
C13	0.0316 (6)	0.0416 (6)	0.0385 (6)	−0.0044 (5)	−0.0045 (5)	−0.0090 (5)

### Geometric parameters (Å, °)

**Table:**

F1—C1	1.3555 (15)	C3—H3A	0.9500
O1—N4	1.2343 (15)	C4—C5	1.3785 (19)
O2—N4	1.2161 (15)	C4—H4A	0.9500
O3—N3	1.2204 (16)	C5—C6	1.3967 (17)
O4—N3	1.2225 (15)	C5—H5A	0.9500
N1—C7	1.2724 (16)	C6—C7	1.4618 (17)
N1—N2	1.3653 (15)	C7—H7A	0.9500
N2—C8	1.3548 (16)	C8—C9	1.4131 (17)
N2—H2A	0.8800	C8—C13	1.4181 (16)
N3—C11	1.4463 (16)	C9—C10	1.3863 (16)
N4—C9	1.4500 (15)	C10—C11	1.3685 (17)
C1—C2	1.3783 (19)	C10—H10A	0.9500
C1—C6	1.3800 (17)	C11—C12	1.3916 (18)
C2—C3	1.381 (2)	C12—C13	1.3597 (18)
C2—H2B	0.9500	C12—H12A	0.9500
C3—C4	1.379 (2)	C13—H13A	0.9500
			
C7—N1—N2	117.04 (11)	C1—C6—C5	116.70 (11)
C8—N2—N1	117.99 (10)	C1—C6—C7	121.17 (11)
C8—N2—H2A	121.0	C5—C6—C7	122.12 (11)
N1—N2—H2A	121.0	N1—C7—C6	118.51 (11)
O3—N3—O4	123.15 (12)	N1—C7—H7A	120.7
O3—N3—C11	117.69 (12)	C6—C7—H7A	120.7
O4—N3—C11	119.16 (11)	N2—C8—C9	123.98 (11)
O2—N4—O1	122.32 (11)	N2—C8—C13	119.48 (11)
O2—N4—C9	119.24 (11)	C9—C8—C13	116.55 (11)
O1—N4—C9	118.44 (11)	C10—C9—C8	121.82 (10)
F1—C1—C2	118.20 (12)	C10—C9—N4	115.72 (11)
F1—C1—C6	118.24 (11)	C8—C9—N4	122.45 (11)
C2—C1—C6	123.55 (12)	C11—C10—C9	118.91 (11)
C1—C2—C3	118.00 (13)	C11—C10—H10A	120.5
C1—C2—H2B	121.0	C9—C10—H10A	120.5
C3—C2—H2B	121.0	C10—C11—C12	121.27 (11)
C4—C3—C2	120.60 (14)	C10—C11—N3	119.78 (11)
C4—C3—H3A	119.7	C12—C11—N3	118.93 (11)
C2—C3—H3A	119.7	C13—C12—C11	119.89 (11)
C5—C4—C3	120.00 (13)	C13—C12—H12A	120.1
C5—C4—H4A	120.0	C11—C12—H12A	120.1
C3—C4—H4A	120.0	C12—C13—C8	121.44 (11)
C4—C5—C6	121.14 (12)	C12—C13—H13A	119.3
C4—C5—H5A	119.4	C8—C13—H13A	119.3
C6—C5—H5A	119.4		
			
C7—N1—N2—C8	−178.38 (11)	N2—C8—C9—N4	2.32 (19)
F1—C1—C2—C3	−179.24 (13)	C13—C8—C9—N4	−177.74 (11)
C6—C1—C2—C3	−0.3 (2)	O2—N4—C9—C10	−6.98 (17)
C1—C2—C3—C4	0.2 (2)	O1—N4—C9—C10	173.40 (12)
C2—C3—C4—C5	0.0 (2)	O2—N4—C9—C8	173.56 (12)
C3—C4—C5—C6	−0.1 (2)	O1—N4—C9—C8	−6.07 (19)
F1—C1—C6—C5	179.17 (11)	C8—C9—C10—C11	0.07 (18)
C2—C1—C6—C5	0.3 (2)	N4—C9—C10—C11	−179.40 (11)
F1—C1—C6—C7	−0.39 (18)	C9—C10—C11—C12	−2.71 (19)
C2—C1—C6—C7	−179.28 (13)	C9—C10—C11—N3	176.08 (11)
C4—C5—C6—C1	0.0 (2)	O3—N3—C11—C10	−173.46 (14)
C4—C5—C6—C7	179.51 (12)	O4—N3—C11—C10	6.06 (19)
N2—N1—C7—C6	178.86 (10)	O3—N3—C11—C12	5.4 (2)
C1—C6—C7—N1	173.99 (12)	O4—N3—C11—C12	−175.12 (12)
C5—C6—C7—N1	−5.54 (19)	C10—C11—C12—C13	2.3 (2)
N1—N2—C8—C9	177.09 (11)	N3—C11—C12—C13	−176.51 (12)
N1—N2—C8—C13	−2.85 (18)	C11—C12—C13—C8	0.8 (2)
N2—C8—C9—C10	−177.12 (11)	N2—C8—C13—C12	176.68 (12)
C13—C8—C9—C10	2.82 (18)	C9—C8—C13—C12	−3.3 (2)

### Hydrogen-bond geometry (Å, °)

**Table:**

*D*—H···*A*	*D*—H	H···*A*	*D*···*A*	*D*—H···*A*
N2—H2A···O1	0.88	2.02	2.6317 (15)	126.
N2—H2A···O1^i^	0.88	2.51	3.3424 (15)	158.
C2—H2B···F1^ii^	0.95	2.45	3.3386 (17)	156.
C3—H3A···O4^iii^	0.95	2.48	3.3177 (19)	148.
C5—H5A···O3^iv^	0.95	2.43	3.2694 (17)	148.

Symmetry codes: (i) −*x*+1, −*y*+1, −*z*+1; (ii) −*x*+3, −*y*, −*z*+1; (iii) *x*+2, *y*−1, *z*; (iv) −*x*+1, −*y*, −*z*.

### Table 2 Cg···Cg π-ring stacking interactions, Cg1 and Cg2 are the centroids of rings C1–C6 and C8–C13; [Symmetry codes: (i) 1+x,y,z; (ii) -1+x, y, z]

**Table:**

*CgI···CgJ*	*Cg···Cg* (Å)	Cg I_Perp (Å)	CgJ_Perp (Å)
Cg1···Cg2^i^	3.6916 (10)	-3.4632 (6)	3.3267 (5)
Cg2···Cg1^ii^	3.6916 (10)	3.3267 (5)	-3.4632 (6)

## References

[bb1] Baughman, R. G., Martin, K. L., Singh, R. K. & Stoffer, J. O. (2004). *Acta Cryst.* C**60**, o103–o106. 10.1107/S0108270103027197 14767127

[bb2] Bolte, M. & Dill, M. (1998). *Acta Cryst.* C**54**, IUC9800065.

[bb3] Fan, Z., Shan, S., Hu, W.-X. & Xu, D.-J. (2004). *Acta Cryst.* E**60**, o1102–o1104.

[bb4] Jasinski, J. P., Guild, C. J., Chidan Kumar, C. S., Yathirajan, H. S. & Mayekar, A. N. (2010). *Acta Cryst.* E**66**, o2832–o2833. 10.1107/S1600536810037980 PMC3008979 21589019

[bb5] Ji, N.-N., Shi, Z.-Q., Zhao, R.-G., Zheng, Z.-B. & Li, Z.-F. (2010). *Bull. Korean Chem. Soc.* **31**, 881–886.

[bb6] Kia, R., Fun, H.-K., Etemadi, B. & Kargar, H. (2009). *Acta Cryst.* E**65**, o833–o834. 10.1107/S1600536809009957 PMC2969085 21582553

[bb7] Liang, Z.-P. (2007). *Acta Cryst.* E**63**, o2943.

[bb8] Motherwell, W. D. S. & Ramsay, J. (2007). *Acta Cryst.* E**63**, o4043.

[bb9] Okabe, N., Nakamura, T. & Fukuda, H. (1993). *Acta Cryst.* C**49**, 1678–1680.

[bb10] Oxford Diffraction (2007). *CrysAlis PRO* and *CrysAlis RED* Oxford Diffraction Ltd, Abingdon, Oxfordshire, England.

[bb11] Shan, S., Xu, D.-J., Wu, J.-Y. & Chiang, M. Y. (2002). *Acta Cryst.* E**58**, o1333–o1335.

[bb12] Sheldrick, G. M. (2008). *Acta Cryst.* A**64**, 112–122. 10.1107/S0108767307043930 18156677

[bb13] Shi, Z.-Q., Ji, N.-N. & Li, X.-Y. (2008). *Acta Cryst.* E**64**, o2135. 10.1107/S1600536808033357 PMC2959777 21580996

